# Supplementary material for: Novel real number representations in Ising machines and performance evaluation: Combinatorial random number sum and constant division
Source: PLoS One. 2024 Jun 13;19(6):e0304594. doi: 10.1371/journal.pone.0304594 (PMC11175401; doi:10.1371/journal.pone.0304594)
Supplement: Attachment — Submitted filename: RevLetter_endo_plos_fin.pdf [file pone.0304594.s001.pdf]

Response to the Reviewers' Comments

Ref. #. PONE-D-23-25182
Title : Novel real number representations in Ising machines and performance evaluation: Combinatorial random number sum and constant division
Author : Katsuhiro ENDO, Yoshiki MATSUDA, Shu TANAKA and Mayu MURAMATSU

Responding to the review results, we have significantly revised the manuscript. We highly appreciate the reviewers' valuable comments and suggestions for corrections, which make the quality of the paper improved.

Below, we provide our responses to the reviewers' comments. Since two reviewers gave their own comments separately, we have prepared separate answers. For each comment, we first cited reviewers' remarks for reference, and then described how we have responded to those. The responses were reflected in the revised manuscript, in which the corrected or newly added contexts were written in red.

We would be very grateful if our paper could be published in "PLOS ONE"

Best regards,
Katsuhiro ENDO, Yoshiki MATSUDA, Shu TANAKA and Mayu MURAMATSU

[For Journal requirements]

1. Please ensure that your manuscript meets PLOS ONE's style requirements, including those for file naming. The PLOS ONE style templates can be found at https://journals.plos.org/plosone/s/file?id=wjVg/PLOSOne_formatting_sample_main_body.pdf and https://journals.plos.org/plosone/s/file?id=ba62/PLOSOne_formatting_sample_title_authors_affiliations.pdf

[Response 1]

Thank you for pointing out the issue regarding the manuscript style. We have checked the PLOS ONE style requirements you provided and have revised the style of the manuscript. The style revisions have been reflected in the revised manuscript.

2. Thank you for stating the following in the Acknowledgments Section of your manuscript:
"This work was supported by the Council for Science, Technology and Innovation (CSTI), Cross398 ministerial Strategic Innovation Promotion Program (SIP), Materials Integration for Revolutionary Design System of Structural Materials" (Funding agency: JST) and "Photonics and Quantum Technology for Society 5.0" (Funding agency: QST), and JST COI-NEXT Grant Number JPMJPF2221.
This work was supported by JSPS KAKENHI (Grant Numbers JP21K03391, JP23H05447) and JST Grant Number JPMJPF2221. The Human Biology-Microbiome-Quantum Research Center (Bio2Q) is supported by the World Premier International Research Center Initiative (WPI), MEXT, Japan.

This work was supported by JST FOREST Program (Grant Number JPMJFR212K, Japan)."
We note that you have provided funding information that is not currently declared in your Funding Statement. However, funding information should not appear in the Acknowledgments section or other areas of your manuscript. We will only publish funding information present in the Funding Statement section of the online submission form.
Please remove any funding-related text from the manuscript and let us know how you would like to update your Funding Statement. Currently, your Funding Statement reads as follows:
"This work was supported by the Council for Science, Technology and Innovation (CSTI), Cross-ministerial Strategic Innovation Promotion Program (SIP), Materials Integration for Revolutionary Design System of Structural Materials" (Funding agency: JST) and "Photonics and Quantum Technology for Society 5.0" (Funding agency: QST), and JST COI-NEXT Grant Number JPMJPF2221.
This work was supported by JSPS KAKENHI (Grant Numbers JP21K03391, JP23H05447) and JST Grant Number JPMJPF2221. The Human Biology-Microbiome-Quantum Research Center (Bio2Q) is supported by the World Premier International Research Center Initiative (WPI), MEXT, Japan.
This work was supported by JST FOREST Program (Grant Number JPMJFR212K, Japan)."
Please include your amended statements within your cover letter; we will change the online submission form on your behalf.

[Response 2]

We apologize for the funding-related text in the manuscript. We understand that the funding statement section of the online submission form should be filled out, not in the manuscript. In the revised manuscript, the funding statement has been removed. In accordance with your suggestion, we have stated the following in the “Funding statement” section of the online submission form:

"This work was supported by the Council for Science, Technology and Innovation (CSTI), Cross-ministerial Strategic Innovation Promotion Program (SIP), Materials Integration for Revolutionary Design System of Structural Materials" (Funding agency: JST) and "Photonics and Quantum Technology for Society 5.0" (Funding agency: QST), and JST COI-NEXT Grant Number JPMJPF2221. This work was supported by JSPS KAKENHI (Grant Numbers JP21K03391 and JP23H05447) and JST (Grant Number JPMJPF2221). The Human Biology-Microbiome-Quantum Research Center (Bio2Q) is supported by the World Premier International Research Center Initiative (WPI), MEXT, Japan. This work was also supported by JST FOREST Program (Grant Number JPMJFR212K, Japan).".

3. Thank you for stating the following financial disclosure:
"This work was supported by the Council for Science, Technology and Innovation (CSTI), Cross-ministerial Strategic Innovation Promotion Program (SIP), Materials Integration for Revolutionary Design System of Structural Materials" (Funding agency: JST) and "Photonics and Quantum Technology for Society 5.0" (Funding agency: QST), and JST COI-NEXT Grant Number JPMJPF2221.
This work was supported by JSPS KAKENHI (Grant Numbers JP21K03391, JP23H05447) and JST Grant Number JPMJPF2221. The Human Biology-Microbiome-Quantum Research Center (Bio2Q) is supported by the World Premier International Research Center Initiative (WPI), MEXT, Japan.
This work was supported by JST FOREST Program (Grant Number JPMJFR212K, Japan)."

Please state what role the funders took in the study. If the funders had no role, please state: "The funders had no role in study design, data collection and analysis, decision to publish, or preparation of the manuscript."
If this statement is not correct you must amend it as needed.
Please include this amended Role of Funder statement in your cover letter; we will change the online submission form on your behalf.

[Response 3]

We apologize for not describing the roles the funders played in this study. All funders listed played no roles. Therefore, in accordance with your suggestion, we have stated in our cover letter that "The funders played no roles in the study design, data collection and analysis, decision to publish, or the preparation of the manuscript."

4. We note that you have indicated that data from this study are available upon request. PLOS only allows data to be available upon request if there are legal or ethical restrictions on sharing data publicly. For more information on unacceptable data access restrictions, please see http://journals.plos.org/plosone/s/data-availability#loc-unacceptable-data-access-restrictions .
In your revised cover letter, please address the following prompts:
a) If there are ethical or legal restrictions on sharing a de-identified data set, please explain them in detail (e.g., data contain potentially sensitive information, data are owned by a third-party organization, etc.) and who has imposed them (e.g., an ethics committee). Please also provide contact information for a data access committee, ethics committee, or other institutional body to which data requests may be sent.
b) If there are no restrictions, please upload the minimal anonymized data set necessary to replicate your study findings as either Supporting Information files or to a stable, public repository and provide us with the relevant URLs, DOIs, or accession numbers. For a list of acceptable repositories, please see http://journals.plos.org/plosone/s/data-availability#loc-recommended-repositories .
We will update your Data Availability statement on your behalf to reflect the information you provide.

[Response 4]

Thank you for your concern regarding the data availability provisions. There are no ethical or legal constraints on sharing minimal data in our study. Therefore, we have placed minimal data in the following stable and public repository: http://github.com/mmc-research-group/Real-number.

5. We note that you have stated that you will provide repository information for your data at acceptance. Should your manuscript be accepted for publication, we will hold it until you provide the relevant accession numbers or DOIs necessary to access your data. If you wish to make changes to your Data Availability statement, please describe these changes in your cover letter and we will update your Data Availability statement to reflect the information you provide.

[Response 5]

We understand that if our manuscript is accepted, its publication will be withheld until we provide the accession number or DOI necessary to access the data. We have made the data available at the following URL: http://github.com/mmc-research-group/Real-number.

6. Please amend either the title on the online submission form (via Edit Submission) or the title in the manuscript so that they are identical.

[Response 6]

Thank you for pointing this out. We have modified the title of the online submission form to match the title of the manuscript.

[For Reviewer 1]

[Comment 1-1]

Changes in representation for search problems has a history going back to the 1980s. The 'bumps' in the binary representation referred to by the authors, for instance, are known in this literature as 'Hamming cliffs'. The authors make no mention of this prior work.

A few key results in this literature are:

1. The 'no free lunch theorem' [Wolpert and Macready, IEEE Trans. Evol. Comp., 1(1), 67-82, 1997] and special-case corollaries [Whitley, Proc. 1st Ann. Conf. Gen. and Evo. Comp., 726-733, 1999].
2. A link between the advantage of the incremental shifts in a Gray or real representation and local correlations in the objective function ('schema similarity') [Caruana and Schaffer, Mach. Learn. Proc., 153-161, 1988].
3. Identification that the behaviour of a function is often highly scale dependent [Goldberg, Complex Systems, 5, 139-167, 1991], with search (often) rapidly approaching a regime in which Gray-type encoding is profitable, and the associated use of hybrid schemes involving dynamic representations, multi-step evolution, and redundant bits.

The representations proposed by the authors, including the randomised encodings, are similar to those considered in [Cheong et al., IEEE Symp. Found. Comp. Int., 251-258, 2007].

[Response 1-1]

We thank the reviewer for taking the time to review our manuscript and providing a critical assessment of our work. We also thank you for sharing with us various important previous studies on the number representation methods in the field of evolutionary computation, which are relevant to our study. We have reviewed in detail all the references you provided and revised the text with appropriate citations. First, we have added in the introduction section that the "Hamming cliff problem" is known in the field of evolutionary computation as a problem caused by the Hamming distance between close numerical values becoming too large when representing integer or real numerical values as a binary number. We have also added in the introduction that Gray coding, which is the integer number representation to make the Hamming distance between adjacent integer values equal to 1, was proposed for the Hamming cliff problem. In addition, we have clearly stated in the methodology section that there is a study in which random number representation is used in the field of evolutionary computation.

Specifically, the following modifications have been made in the revised manuscript:

added: on page 5, line 97.

“There are important studies on the problem of such real number representations in the context

of evolutionary computing. In this context, the requirement of simultaneous bit flipping of the binary representation mentioned above is known as the Hamming cliff problem [47,48]. The integer number representation called "Gray coding" has been proposed to eliminate the Hamming cliff problem [49,50]. Gray coding is a well-constructed representation in which only one bit is flipped to increase a value by one, and this property improves the optimization efficiency of evolutionary computation."

added: on page 10, line 213.

"Note that there is a previous study of real number representations by summing random numbers in the field of evolutionary algorithms [58]."

[Comment 1-2]
Now, with the above caveats, those aspects of the representation problem specific to the QUBO restriction and so to quantum annealing do remain, to my knowledge, largely unexplored. For instance, do Gray codes necessarily introduce higher order non-linearities that are disallowed in this setting? There appears to be room for discussion here, and I believe the results of the authors could serve to open this discussion, but some acknowledgement of prior, related work is necessary.

[Response 1-2]

Thank you for your positive view of this paper.

As you have pointed out, Gray coding cannot be expressed as a weighted linear sum of bits, but it has higher-order nonlinear relations for each bit. In quantum annealing, it is necessary to convert the problem into a QUBO Hamiltonian (quadratic function of binary variables), so it is not possible to directly embed such a higher-order nonlinear expression. The introduction of auxiliary bits by a method called order reduction can reduce the higher-order terms to second-order terms, but this increases the number of bits required and complicates the problem. Therefore, it is difficult to use Gray coding as a number representation method for quantum annealing. Therefore, we are discussing real number representations only in the range of weighted linear sums. We have added in the introduction of the revised manuscript that Gray coding proposed in the context of evolutionary computation is difficult for the above reasons.

added: on page 5, line 103.

"However, Gray coding cannot be represented as a weighted linear sum of bits, but it has higher-order nonlinear relations for each bit. In quantum annealing, it is necessary to transform the problem into a QUBO Hamiltonian (quadratic function of bits), and such high-order nonlinear terms cannot be directly embedded. Although the order of the Hamiltonian can be reduced by introducing auxiliary bits, the number of bits required increases and the problem becomes more complicated. Therefore, Gray coding is not suitable for quantum annealing, and we have to discuss real number representations in the range of weighted linear sums."

[Comment 1-3]
Otherwise, I believe the analysis in the paper is understandable and credible. The authors might consider slight revision to clarify their error analysis and to introduce some estimates of sample deviation in their plots.

[Response 1-3]

Thank you for your suggestion about the error analysis.

As you pointed out, we have replaced Fig 1 in the original manuscript with the figure below as Fig 2 in the revised manuscript with the error bar added. (The numbering of the figures has changed owing to the addition of a new figure for another reason.) The error bars indicate the standard error (SE) of the mean of the original samples.

modified: Fig 2 in the revised manuscript (from Fig 1 in the original manuscript).

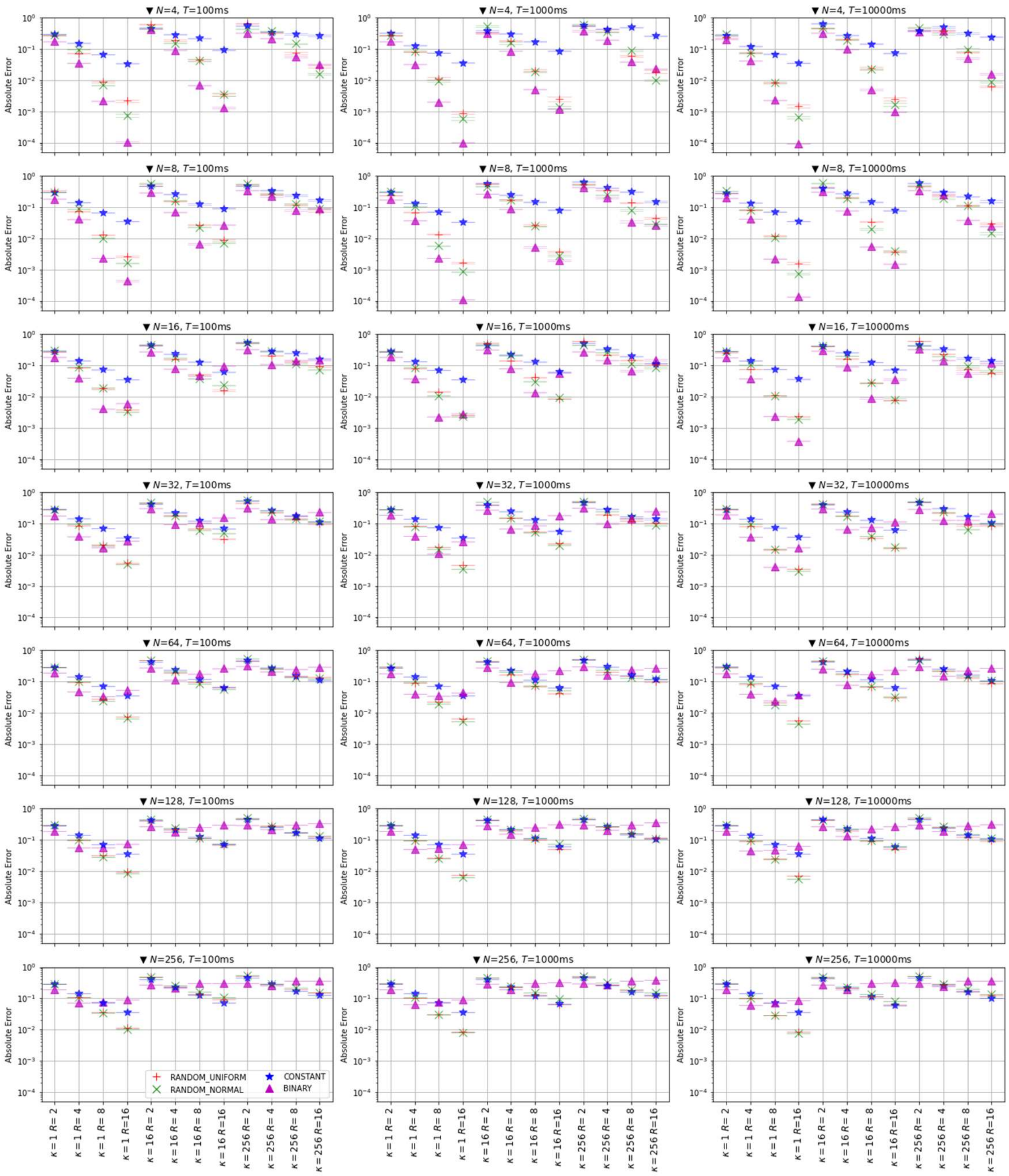

modified: the title of Fig 2

From: “Errors of the four representations, RANDOM_UNIFORM, RANDOM_NORMAL, CONSTANT, and BINARY, when linear equations are solved using Fixstars Amplify AE with various settings.”

To: “Mean errors of the four representations, RANDOM_UNIFORM, RANDOM_NORMAL, CONSTANT, and BINARY, when linear equations are solved using the Fixstars Amplify AE with various settings. The error bars denote the standard error of the mean errors.”

[For Reviewer 2]

Overall, this manuscript has many significant flaws, both in the presentation of the information, and the actual experimental details. This manuscript requires extremely extensive revision and justification of the proposed methods to be suitable for publication, and without significant improvement I would recommend rejection of the paper.

We thank the referee for taking the time to review our manuscript and providing a critical assessment of our work. On the basis of your comments and questions, we have performed additional experiments and have updated the manuscript. Please find our responses to your questions below.

# General Notes:

[Comment 2-1]
Figures 2, 3, 4, 5 do not have axis labels! They absolutely need axis labels for the figures to be understandable.

[Response 2-1]

We apologize for forgetting to include the axis labels on each figure. We have added the axis labels to these figures in the revised manuscript.

modified: Figs 3, 4, 5, and 6 in the revised manuscript (from Figs 2, 3, 4, and 5 in the original manuscript, respectively).

(Note: We did not include the modified figures here because the reply would be too long if we include all of them here.)

[Comment 2-2]
The primary focus of the paper is around what the authors refer to as "representation of real numbers". I find this particular phrase extremely confusing. The real numbers that are of interest here are the cost function values for discrete combinatorial optimization problems. First, they do not need to be high precision real numbers - they can be integers. But more importantly, I do not think that the proposed methods are representing real numbers in a different way, I think they are representing cost functions in a different way. Which is fine, assuming the cost functions are correct, but this term is very confusing.

[Response 2-2]

Thank you for pointing out that the description "representation of real numbers" is confusing.

Our target is to solve the problem with real variables as decision variables. The term "representation of real numbers" is used in the manuscript because quantum annealing machines cannot

handle real numbers directly and real numbers should be represented by some combination of binary variables.

However, as the reviewer stated, using the real number representation for a cost function in which the real variables are the decision variables would lead to a discrete optimization problem in which the binary variables are the decision variables (approximated with finite precision, as the reviewer also stated). Therefore, we are in fact investigating the properties of different discrete optimization problems caused by different real number representations. Thus, on the basis of the reviewer's comments, we have modified the text to clarify this point as follows.

added: on page 8, line 164.

"This Hamiltonian takes different forms when different real number representations are used. That is, different real number representations transform the optimization problem in Eq. (1) into different types of discrete optimization problem."

[Comment 2-3-1]
Following from the previous point, lines 38-41 state that "we noticed that when real numbers are represented in binary number, there are numbers that can only be reached by inverting several bits simultaneously under the restriction of not increasing a given Hamiltonian, which makes the optimization very difficult.". I do not understand what is being said here -- what does it mean that real numbers are represented in "binary number". For optimization, there is a cost function that defines a mapping from a set of variables to some cost output, which is a real number. But, that does not mean that the real numbers are represented in binary, rather the binary variables have some cost defined by them depending on what cost function you care about. *[Note: We have split your comment for the sake of a clear response. The second half of your comment is in the next section.]*

[Response 2-3-1]

We apologize for the insufficient explanation regarding the meaning of expressing real numbers in binary numbers.

To clearly explain the representation of real numbers in binary variables, we have drawn a schematic diagram (Fig 1 in the revised manuscript) that shows the process of solving the linear equation problems with the real number representation (including binary number representation).

In Fig. 1 in the revised manuscript, we first have a problem to solve. In this study, the problem to be solved are linear equations with real variables as the decision variables. The linear equations are then converted to the unconstrained problem using the least-squares form. This unconstrained problem can be transformed into a QUBO Hamiltonian by representing the real variables as a weighted linear sum of several binary variables. We call this method of representing real variables as a combination of binary variables the "real number representation". Then, the original problem can be solved by solving the optimal assignment of the binary variables in QUBO with Ising machines and restoring this assignment to a real number solution of the original problem based on the real number representation.

Therefore, the "representation of real numbers in binary numbers" is one method of real number representation for converting problems with real numbers as decision variables to QUBO Hamiltonians. That is, the binary number representation is a representation of a real number by assigning a binary variable to each digit of the corresponding base-2 real number representation.

We have added the schematic diagram (Fig 1) and its explanations to the main text for easier understanding of the explanation.

added: Fig 1 in the revised manuscript.

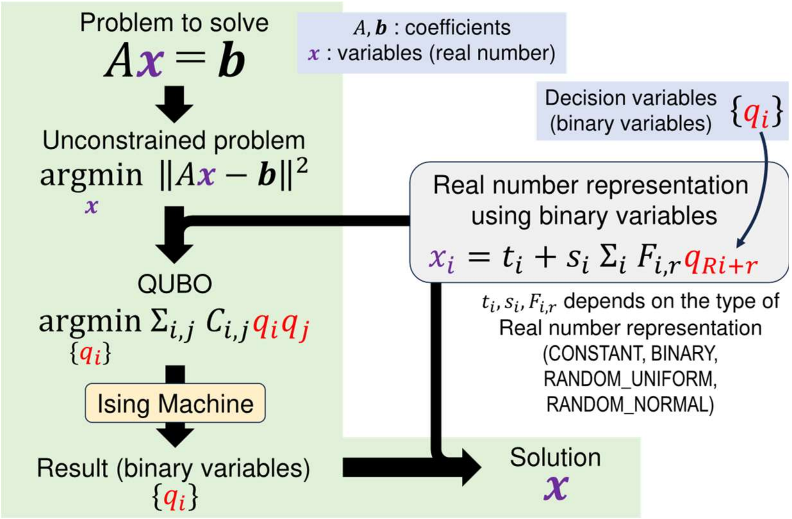

added: the title of Fig 1

"Schematic diagram showing the process of solving the linear equations by Ising machines."

added: on page 8, line 170.

"The output of the Ising machine is finally transformed to a real value solution of the original problem in Eq. (1) on the basis of the the real number representation used. All of the above methods are summarized in Fig 1."

[Comment 2-3-2]

Following from the previous point, lines 38-41 state that "we noticed that when real numbers are represented in binary number, there are numbers that can only be reached by inverting several bits simultaneously under the restriction of not increasing a given Hamiltonian, which makes the optimization very difficult.". I do not understand what is being said here -- what does it mean that real numbers are represented in "binary number". For optimization, there is a cost function that defines a mapping from a set of variables to some cost output, which is a real number. But, that does not mean that the real numbers are represented in binary, rather the binary variables have some cost defined by them depending on what cost function you care about. Also, why is the restriction of fewer bitflips relevant? Especially for quantum annealing, early on the anneal when the transverse field is dominating the states of the qubits are flipping very often. Additionally, bit assignments to get to a lower energy state do not necesarely need to be inverted all at the "same time".

[Response 2-3-2]

We apologize for the insufficient explanation of why the restriction of fewer bit flips is relevant.

We have added new arguments and calculation results in the revised manuscript to reinforce the explanations as follows. We hope you will review them.

As a premise, the part you pointed out (lines 38–41) in the original manuscript is described in terms of the simulated annealing on classical computers, as shown at the beginning of the sentence on line 38 in the original manuscript ("Considering the classical annealing point of view, we noticed that ..."). Thus, let me first explain what happens in the simulated annealing.

The simulated annealing algorithm searches through the neighborhood to reach the optimal state. When we assume that the optimization problem has the decision variable with integer (or real) values and these values are expressed in some integer (or real) number representations (for example, 7 is represented as [0111] and 8 is represented as [1000] for the integer version of binary number representations), the neighborhood state is usually defined as the state generated by inverting one bit of a current state (e.g., [0100] ⇔ [0101] are neighborhood states).

Since the simulated annealing algorithm preferentially searches for states with decreasing cost function values, it is difficult to reach the optimal state if the cost function value does not decrease unless many bits are flipped simultaneously and if the optimal state is at the end of flipping such bits. Here, as noted in the original manuscript (line 87), when using binary number representation, if the current state is 7 [0111] and the distance from 8 [1000] is defined as the cost function, a one-bit reversal (= transition to one of the neighborhood states) will always result in a worse cost function value. That is, to improve the value of the cost function from the current state (7 [0111]), several bits must be flipped at the same time, and this is not a neighbor state. On the other hand, using the integer version of the constant representation, the cost function can be improved by flipping one bit (e.g., 7 [1101111111] ⇒ 8 [1111111111]). This is why the restriction of fewer bit flips is relevant and why we believe that, at least when using the simulated annealing, the accuracy of the obtained solution differs depending on the real number representation.

However, as the reviewer stated, it is not immediately clear whether this difference in real number representation also occurs in quantum computers. While we claim that our D-wave hybrid solver results (Fig 3 in the original manuscript; Fig 4 in the revised manuscript) indirectly suggest the same trends, we have added a new direct analysis to the revised manuscript that shows such a difference can also occur in quantum annealing machines. Fig 7 shows the results of the new analysis.

In Fig. 7, we show the results of solving the following problem by the simulation of quantum annealing. First, we considered a single real number as a decision variable x and a cost function as the squared distance from a target real value y, $L = (x - y)$^2. We assume that the decision variable x ranges from 0 to 1 and that the target real value y is chosen from a uniform distribution in the range from 0 to 1. The optimal value of x for this problem is obviously $x = y$. To solve this problem using quantum annealers, x is represented as R binary variables $\{q_i\}$ $(i = 1, \ldots, R)$ using some kind of real number representation ($x = t + s\, \Sigma\, F_i q_i$), and then the cost function takes the QUBO form, $L = (x - y)$^2 $= (t + s\, \Sigma\, F_i q_i\ - y)$^2.

To find the optimal value of x of this QUBO problem the following simulation of quantum annealing on classical computers was performed. First, to convert the obtained QUBO Hamiltonian into a quantum Hamiltonian on quantum computers, we replace q_i with $(1 - Z_i)/2$ for each binary variable q_i to obtain the quantum Hamiltonian H_{Z}, which is a $2^R \times 2^R$ Hermitian matrix. Here, Z_i is the Pauli Z matrix on the i-th qubit. The quantum annealing machine can generate the ground state of this quantum Hamiltonian H_{Z}, which corresponds to the optimal solution of the QUBO problem.

Next, we simulate the quantum annealing process of the quantum annealing machine to find the ground state of this quantum Hamiltonian H_{Z}. We define a time-dependent Hamiltonian $H(t)$ =

$(1-t/T)H_X + (t/T)\,H_Z$ that varies in time from the transverse field Hamiltonian $H_X = \Sigma X_i$ to the target Hamiltonian H_Z. Here, X_i is the Pauli X matrix on the i-th qubit and T is the time taken for the Hamiltonian to change over time. According to the quantum adiabatic theorem, which is the theoretical basis of quantum annealing, a quantum state that was the ground state of the initial Hamiltonian $H(t=0)$ at the initial time remains in the ground state of the Hamiltonian at each time, $H(t)$, if the Hamiltonian changes with time slowly enough. We therefore simulate the time development of the quantum state $\varphi(t)$ under the time-dependent Hamiltonian $H(t)$, $d\varphi(t)/dt = -iH(t)\varphi(t)$, with the ground state of the Hamiltonian H_X as the initial quantum state $\varphi(t=0)$. This simulates the actual computational process in a quantum annealing machine without any noise.

To simulate this quantum time evolution on classical computers, we compute $\varphi(t+\Delta t) = \exp\{-i\Delta t H(t)\}\,\varphi(t)$ from $t=0$ to T to obtain the final quantum state $\varphi(T)$, which is one of the explicit finite-difference methods. Here, Δt is the time step, and T=1.0 and $T/\Delta t$=10^4 were used in this experiment, in which we have confirmed that this number of divisions $T/\Delta t$ is sufficient for the calculation accuracy.

Note that when T is sufficiently large, the final state $\varphi(T)$ should coincide with the ground state of the Hamiltonian H_Z from the quantum adiabatic theorem. However, we are not interested in the case where T is sufficiently large. This is because the BINARY representation, which has the highest representational capability for real numbers, should yield the best results if the optimal state of the QUBO problem is obtained. Instead, what we are interested in is the difference in the results that different real number representations yield a realistically small T.

If T is not sufficiently large, the accuracy that can realistically be achieved by using each real number representation can be examined. Here, if T is not sufficiently large, the final state $\varphi(T)$ does not coincide with the optimal state but is a superposition of multiple states. When such final state $\varphi(T)$ is measured, the measurement result and the solution x derived from it vary stochastically. Thus, we can calculate the probability p that the error between the given result x obtained from the bits measured using real number representation and the correct answer y, $|x-y|$, is smaller than the given threshold E. In other words, the error probability function $E(p)$ can be defined to indicate that the error E can be achieved with the probability p. Here, note that the error probability function $E(p)$ also depends on the randomness of the selected the target value y and the randomness of the real number representation. To account for this randomness, the average $E(p)$ for 1000 independent trials, $\langle E(p)\rangle$, is calculated for statistical comparison. This averaged error probability functions $\langle E(p)\rangle$ is plotted in Fig 7, in which the results are shown in different panels for different numbers of binary variables used in the real number representation, R. The differences in the real number representation are indicated by different curves in each inset.

Fig 7 shows that the statistical trends of the results differ significantly depending on the real number representation. First, in the BINARY representation, the error is smaller for a small p than in the other real number representations. This result is consistent with the fact that the BINARY representation has the highest representational capability.

On the other hand, for a large p in the BINARY representation, the error deteriorates more rapidly than for the other real number representations, and the error is clearly larger than for the other methods especially when the number of bits used is 6 or more. This result is also consistent with the experimental results (Figs 2–4), which show that the BINARY representation has high representational power but fails to take advantage of that power and loses to other real-number representations when a

large number of bits are used.

Next, the CONSTANT representation has the largest error at a low probability p owing to its limited expressive power, but the smallest error is achieved at a high probability p. Moreover, the two RANDOM representations are a good mix of the BINARY and CONSTANT representations because they have smaller errors than the BINARY representation at low probabilities and smaller errors than CONSTANT at high probabilities. These results are also consistent with the experimental results.

Therefore, this task shows that as in the case of simulated annealing, the difference in real number representation has also a significant impact on quantum annealing. We believe that these results indicate that the arguments discussed in Tasks 1 and 2 regarding the number of simultaneous bit flips required to reduce energy in classical simulated annealing are also important in quantum annealing.

To address the insufficient explanation of why the restriction of fewer bit flips is relevant, we have added Fig 7 to the main text and have revised the text to add an explanation of the new task as follows:

added: Fig 7 in the revised manuscript.

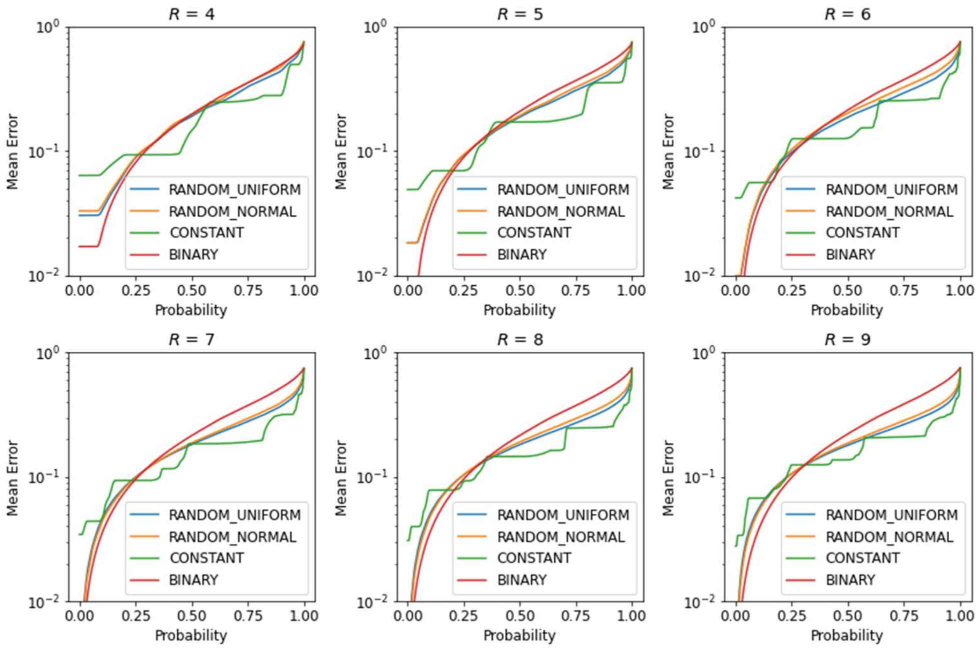

added: the title of Fig 7

"Results of Task 3. The figure shows panels for different number of bits, R. Each curve indicates the averaged error probability function $\langle E(p) \rangle$."

added: on page 19, line 419.

"Task 3 In Task 1 and 2, we examined the efficiency of bit-flip-based search, which mimics the process of simulated annealing when using different real number representations. However, it is not certain that quantum annealing machines have the same properties. Therefore, in Task 3, we discuss the properties of quantum annealing machines by performing a task that directly simulates the process of quantum annealing using a simple optimization problem."

added: on page 19, line 426.

"In Fig. 7, we show the results of solving the following problem by the simulation of quantum annealing. First, we considered a single real number as a decision variable x and a cost function as the squared distance from a target real value y, $L = (x - y)\text{^}2$. We assume that the decision variable x ranges from 0 to 1 and that the target real value y is chosen from a uniform distribution in the range from 0 to 1. The optimal value of x for this problem is obviously $x = y$. To solve this problem using quantum annealers, x is represented as R binary variables $\{q_i\}$ $(i = 1, \ldots, R)$ using some kind of real number representation ($x = t + s\,\Sigma\, F_i q_i$), and then the cost function takes the QUBO form, $L = (x - y)\text{^}2 = (t + s\,\Sigma\, F_i q_i\ - y)\text{^}2$.

To find the optimal value of x of this QUBO problem the following simulation of quantum annealing on classical computers was performed. First, to convert the obtained QUBO Hamiltonian into a quantum Hamiltonian on quantum computers, we replace q_i with $(1 - Z_i)/2$ for each binary variable q_i to obtain the quantum Hamiltonian H_{Z}, which is a $2^R \times 2^R$ Hermitian matrix. Here, Z_i is the Pauli Z matrix on the i-th qubit. The quantum annealing machine can generate the ground state of this quantum Hamiltonian H_{Z}, which corresponds to the optimal solution of the QUBO problem.

Next, we simulate the quantum annealing process of the quantum annealing machine to find the ground state of this quantum Hamiltonian H_{Z}. We define a time-dependent Hamiltonian $H(t) = (1 - t/T)H_X\ + (t/T)\ H_{\mathrm{Z}}$ that varies in time from the transverse field Hamiltonian $H_{\mathrm{X}} = \Sigma\, X_i$ to the target Hamiltonian H_{Z}. Here, X_i is the Pauli X matrix on the i-th qubit and T is the time taken for the Hamiltonian to change over time. According to the quantum adiabatic theorem, which is the theoretical basis of quantum annealing, a quantum state that was the ground state of the initial Hamiltonian $H(t = 0)$ at the initial time remains in the ground state of the Hamiltonian at each time, $H(t)$, if the Hamiltonian changes with time slowly enough. We therefore simulate the time development of the quantum state $\varphi(t)$ under the time-dependent Hamiltonian $H(t)$, $d\varphi(t)/dt = -iH(t)\varphi(t)$, with the ground state of the Hamiltonian H_{X} as the initial quantum state $\varphi(t = 0)$. This simulates the actual computational process in a quantum annealing machine without any noise.

To simulate this quantum time evolution on classical computers, we compute $\varphi(t + \Delta t) = \exp\{-i\Delta t H(t)\}\,\varphi(t)$ from $t = 0$ to T to obtain the final quantum state $\varphi(T)$, which is one of the explicit finite-difference methods. Here, Δt is the time step, and T=1.0 and $T/\Delta t$=10^4 were used in this experiment, in which we have confirmed that this number of divisions $T/\Delta t$ is sufficient for the calculation accuracy.

Note that when T is sufficiently large, the final state $\varphi(T)$ should coincide with the ground state of the Hamiltonian H_{Z} from the quantum adiabatic theorem. However, we are not interested in the case where T is sufficiently large. This is because the BINARY representation, which has the highest representational capability for real numbers, should yield the best results if the optimal state of the QUBO problem is obtained. Instead, what we are interested in is the difference in the results that different real number representations yield a realistically small T.

If T is not sufficiently large, the accuracy that can realistically be achieved by using each real number representation can be examined. Here, if T is not sufficiently large, the final state $\varphi(T)$ does not coincide with the optimal state but is a superposition of multiple states. When such final state $\varphi(T)$ is measured, the measurement result and the solution x derived from it vary stochastically. Thus, we can calculate the probability p that the error between the given result x obtained from the bits

measured using real number representation and the correct answer y, $|x - y|$, is smaller than the given threshold E. In other words, the error probability function $E(p)$ can be defined to indicate that the error E can be achieved with the probability p. Here, note that the error probability function $E(p)$ also depends on the randomness of the selected the target value y and the randomness of the real number representation. To account for this randomness, the average $E(p)$ for 1000 independent trials, $\langle E(p) \rangle$, is calculated for statistical comparison. This averaged error probability functions $\langle E(p) \rangle$ is plotted in Fig 7, in which the results are shown in different panels for different numbers of binary variables used in the real number representation, R. The differences in the real number representation are indicated by different curves in each inset.

Fig 7 shows that the statistical trends of the results differ significantly depending on the real number representation. First, in the BINARY representation, the error is smaller for a small p than in the other real number representations. This result is consistent with the fact that the BINARY representation has the highest representational capability.

On the other hand, for a large p in the BINARY representation, the error deteriorates more rapidly than for the other real number representations, and the error is clearly larger than for the other methods especially when the number of bits used is 6 or more. This result is also consistent with the experimental results (Figs 2–4), which show that the BINARY representation has high representational power but fails to take advantage of that power and loses to other real-number representations when a large number of bits are used.

Next, the CONSTANT representation has the largest error at a low probability p owing to its limited expressive power, but the smallest error is achieved at a high probability p. Moreover, the two RANDOM representations are a good mix of the BINARY and CONSTANT representations because they have smaller errors than the BINARY representation at low probabilities and smaller errors than CONSTANT at high probabilities. These results are also consistent with the experimental results.
Therefore, this task shows that as in the case of simulated annealing, the difference in real number representation has also a significant impact on quantum annealing. We believe that these results indicate that the arguments discussed in Tasks 1 and 2 regarding the number of simultaneous bit flips required to reduce energy in classical simulated annealing are also important in quantum annealing."

[Comment 2-4]
The authors state that experiments were performed on D-Wave quantum annealers, and "Fixstars Amplify Annealing Engine". First, the exact names (e.g. chip ids in the case of the D-Wave quantum annealers) needs to be specified. Second, the technology of the devices needs to be described. For example, were any hybrid quantum annealing solvers used? What what is the technology behind "Fixstars Amplify Annealing Engine"? Much of the paper descrbed quantum annealing technology, but is "Fixstars Amplify Annealing Engine" a digital annealer, simulated annealing, some other type of classical optimization technique (and who is the manufacturer)? Third, the solver parameters used need to be very explicitely stated for all machines - for example, for the D-Wave quantum annealers was minor-embedding used, what annealing time was used, if minor embeddings were used what was the structure and number of variables, and what chain break resolution algorithm was used?

[Response 2-4]

We apologize for the lack of explanation of the device settings.

In our experiments, we used the following three annealing devices: (1) Fixstars Amplify

Annealing Engine, a GPU-based simulated annealing machine developed by Fixstars amplify, (2) hybrid_binary_quadratic_model_version2, a hybrid solver developed by D-wave, and (3) Advantage_system1.1, a full quantum solver developed by D-wave.

In the experiments described in the main text, we used the Amplify SDK middleware to enable unified handling across digital annealers, hybrid solvers, and full-quantum solvers. Amplify SDK can use Fixstars Amplify Annealing Engine owned by Fixstars Amplify, Advantage/Leap Hybrid Solver by D-wave, and others by changing the settings of the target computing environment.

In all of our experiments use the default settings of Amplify SDK, so we did not configure the devices in additional settings. Thus, we were not aware of the configuration items you mentioned, as they depend on the internal implementation of Amplify SDK.

To address your point, we contacted the developer, Fixstars amplify, regarding the internal settings of Amplify SDK and we obtained the following information. When using Advantage_system1.1, the find_clique_embedding function included in D-wave API is used for minor embedding. For the chain break resolution algorithm, the majority vote is used, which is the standard method in D-Wave's library. When using hybrid_binary_quadratic_model_version2, the QUBO data is internally transferred to the LeapHybridSolver of D-wave API without any additional configuration; therefore, no configuration parameters exist except annealing time, which we described in the original manuscript.

We have added the above device information and device settings to the revised manuscript. Specifically, we have made the following modifications.

added: on page 12, line 261.

"In the experiment, we used the Fixstars Amplify Annealing Engine, a GPU-based simulated annealing machine, and the Amplify SDK middleware developed by Fixstars amplify."

added: on page 15, line 319.

"As a hybrid solver, we used hybrid_binary_quadratic_model_version2 via the Amplify SDK middleware. As a full quantum solver, we used Advantage_system1.1 via the Amplify SDK middleware. In this case, The Amplify SDK middleware uses find_clique_embedding function provided by D-wave API for minor embedding and the majority vote for the chain break resolution algorithm."

[Comment 2-5]
Line 151-152 states that "Since the longer the computation time, the better the expected solution, the timeout time". Well, this is not strictly true for all problem types in the case of quantum annealing.

[Response 2-5]

Thank you for pointing out that a longer the computation time does not always lead to better solutions.

According to the quantum adiabatic theorem, a sufficiently long annealing time will always yield the optimal state for a noiseless quantum annealing machine. However, if the annealing time is insufficient, it is unclear whether increasing the annealing time facilitates the achievement of a better state, as you pointed out.

Therefore, we examined the effect of increasing the annealing time on a simple example. Specifically, in the task of performing an ideal quantum annealing for one real number problem

introduced in Comment 3-2, we plotted in Fig EX1 the trend of error probability function <E(p)> changes when the annealing time is increased tenfold from the original T=1.0 (Fig 7 in the revised manuscript). Comparing Figure 7 and Figure EX1 shows that the error probability function shifts downward as the annealing time increases. This represents a significant increase in the probability of achieving a given error, indicating that the increase in annealing time improved the solution. This result does not mean that increasing annealing time has a positive effect on all problems, and in actual machines, it may worsen owing to decoherence and other factors. Therefore, we have modified the text to state that the annealing time can be correlated with the accuracy of the solution considering the quantum adiabatic theorem as follows:

modified: on page 8, line 172.

From: “Since the longer the computation time, the better the expected solution, the timeout time T at which the machines stop performing is one of the parameters that should be considered.”

To: “In ideal quantum annealers, according to the quantum adiabatic theorem, the optimal state is always obtained if the annealing process takes a sufficiently long time. Thus, we can consider that in an ideal noiseless quantum computer, the longer the annealing time, the better the quality of the solution. Therefore, it is necessary to consider the timeout time T as a parameter that controls the accuracy of the solutions.”

Fig EX1: (only for the reviewers)

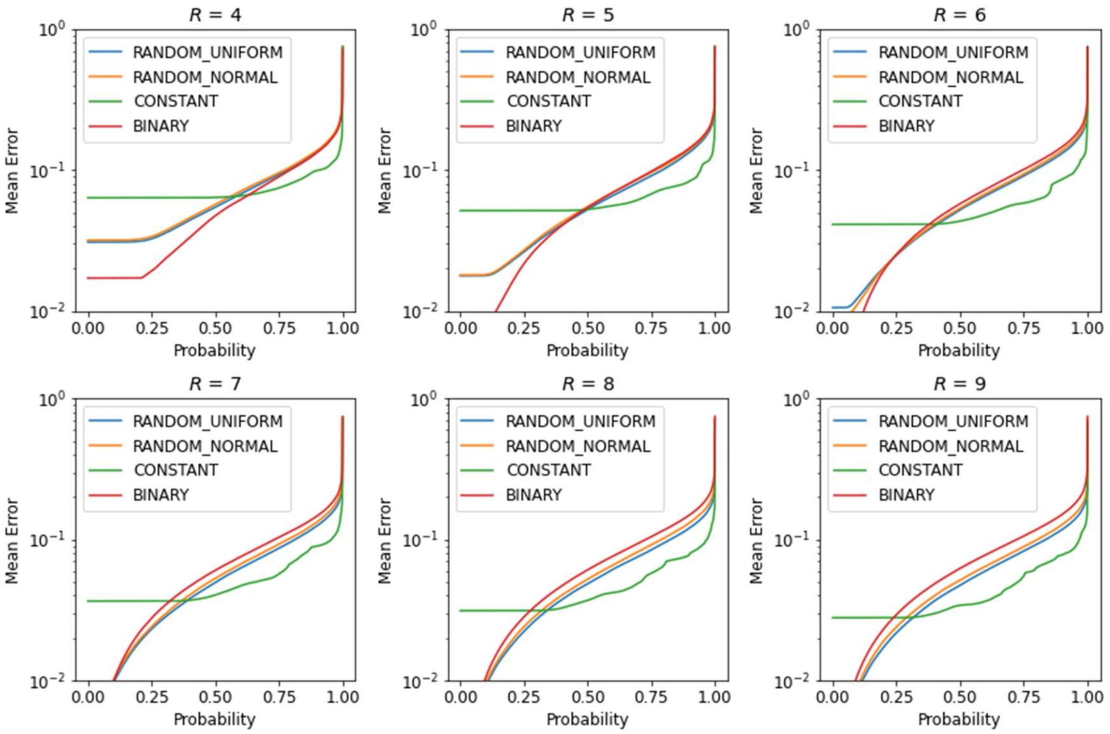

[Comment 2-6]

Something to note is that D-Wave quantum annealers have limited precision for programming the

coefficient weights on the hardware, so the uniform random coefficient choices will mean that the quantum annealers are unlikely to find the global ground state, especially if minor embedding was used (I assume it was).

[Response 2-6]

Thank you for pointing out that the limited accuracy of the coefficient weights on the D-wave machine may prevent the achievement of the correct global ground state. As you pointed out, the precision of the coefficient weights may indeed be insufficient when using a binary or random number representation, which generates small coefficient weights.

In the revised manuscript, we have described the possibility that the limited precision may be the reason why we did not obtain results that were well consistent with the other results in the case of full quantum experiments. Specifically, we have revised the following.

modified: on page 16, line 337.

From: "For the full quantum solver in Fig. 3b, however, there is no significant difference between the methods, although this may be due to the insufficient scale of the programs."

To: "For the full quantum solver in Fig 4B, however, there is no significant difference between the methods. This could be due to the insufficient size of the program or the limited precision of the coefficient weights of the D-Wave hardware, including the effect of minor embeddings."

[Comment 2-7]

Is equation 5 applied iteratively in order to compute better and better x solutions?

[Response 2-7]

As shown in the schematic diagram in Fig 1 in the revised manuscript (which is newly added in the revised manuscript), a single QUBO Hamiltonian (Equation 5 in the manuscript) is generated from the linear equations to be solved. The solution to the linear equations can be found by performing a single run of annealing to find the best combinations of binary variables for which the Hamiltonian in Equation 5 has a minimum value. In simulated annealing (e.g. Fixstars Amplify Annealing Engine), Equation 5 is classically computed repeatedly to calculate the value of the Hamiltonian for different states.

We hope the above has sufficiently answered your question.

[Comment 2-8]

What is the matrix size that corresponds to the varying number of variables (line 237)? I assume it is the square root of N

[Response 2-8]

In this study, as stated around Equation 1 in the original manuscript, we consider only the case where the number of equations in the linear equations coincides with the number of unknown real variables. Therefore, for the number of real variables N, the matrix size of the matrix A is $N \times N$.

We hope that above has sufficiently answered your question.

[Comment 2-9]

Is q the decision variable in equations 3 and 4?

[Response 2-9]

Yes it is.

As we mentioned in our response to comment 3-1, we apologize for the lack of explanation about the decision variables. As shown in Fig 1, which is a schematic diagram of our process to solve linear equations, the binary variables $\{q_i\}$ are the decision variables to represent unknown real values of the linear equations. Note also that there are no decision variables other than the binary variables used to represent the real numbers of variables in the linear equations.

We hope that the above has sufficiently answered your question.

[Comment 2-10]

Line 147 and equation 5; I would like to see a more explicit example of one of these QUBOs -- I think that almost always QUBOs constructed for matrix factorization will yield higher order terms.

[Response 2-10]

For the sake of clarity, we should have written an explicit form of QUBO.

The explicit QUBO of the Hamiltonian can be obtained by substituting a real number representation with binary variables (Equation 4) for Equation 5 in the original manuscript. Since Equation 4 is a first-order form for binary variables, substituting it into Equation 5 in the original manuscript results in a second-order form, and no higher-order terms arise. More specifically, the following QUBO is generated:

$$H = \sum_{i=0}^{N-1}\left(b_i - \sum_{j=0}^{N-1} A_{ij}x_j\right)^2 = \sum_{i=0}^{N-1}\left(b_i - \sum_{j=0}^{N-1} A_{ij}\left(t_j + s_j\sum_{r=0}^{R-1} F_{j,r}q_{Rj+r}\right)\right)^2.$$

We have modified Equation 5 as follows to indicate the explicit form of QUBO.

modified: Equation 5 on page 8, line 168.

From:

$$H = \sum_{j=0}^{N-1}\left(b_i - \sum_{j=0}^{N-1} A_{ij}x_j\right)^2.$$

To:

$$H = \sum_{i=0}^{N-1}\left(b_i - \sum_{j=0}^{N-1} A_{ij}x_j\right)^2 = \sum_{i=0}^{N-1}\left(b_i - \sum_{j=0}^{N-1} A_{ij}\left(t_j + s_j\sum_{r=0}^{R-1} F_{j,r}q_{Rj+r}\right)\right)^2.$$

[Comment 2-11]

What are the "real variables x" in line 144? Are these the decision variables? or is x the coefficient of the decision variables?

[Response 2-11]

The real variables $\boldsymbol{x}$ are the decision variables in the linear equations to be solved. However, in this study, real numbers are represented as a combination of binary variables to solve linear equations with Ising machines. Therefore, as shown in Fig 1, once the QUBO Hamiltonian corresponding to the linear equations is generated, the real numbers $\boldsymbol{x}$ are represented by the binary decision variables. In this sense, $\boldsymbol{x}$ are no longer decision variables.

We hope that the above has sufficiently answered your question.

[Comment 2-12]
i is not defined in equation 5

[Response 2-12]

It was a notation error in equation 5. Thank you for pointing this out.

Please find that the modifications for this point are included in the modification of equation 5 in the response to Comment 2-10.

[Comment 2-13]
Line 139; what is {F_i, r} ? Are these two distinct real numbers being multiplied?

[Response 2-13]

As noted around Equation 4 in the original manuscript, $\{F_{i,r}\}$ are the mapping coefficients for representing the real numbers $\boldsymbol{x} = \{x_i\}$ in binary variables. In Equation 4, the real number x_i is represented as $x_i = t_i + s_i \sum_{r=0}^{R-1} F_{i,r} q_{Ri+r}$; thus, $F_{i,r}$ is the weight of the r-th binary variable in the i-th real number x_i to represent the value of the i-th real number x_i. That is, $\{F_{i,r}\}$ is a set consisting of RN coefficients.

We hope that the above has sufficiently answered your question.

[Comment 2-14]
Critically, the central procedure that this paper describes is completely not clear. Equation 4 appears to be a polynomial containing only linear terms, with decision variables q. It is not clear how the four different "real number representations" are actually applied to the problem QUBO. To me, Equations 7, 8, 9, and 10 appear to just be four different ways of generating coefficients for the linear terms in Equation 4 -- it is completely unclear whether the generated QUBOs (all of which are, it seems, general integer matrix factorization QUBOs) using these four "real number representations" are actually equivalent (e.g. do the QUBOs/ Ising models have the same eigenspectrum, or at least the same ground states). This comparison only works (the central argument of the paper) if the four "real number representation" methods are actually producing equivalent QUBOs.

[Response 2-14]

Thank you for pointing out the non-equivalence of QUBO in different real number representations.

As shown in Figure 1 in the revised manuscript, our research is to investigate how the accuracy of the solution of linear equations is affected by using different representations of real numbers when solving problems using Ising machines. As you have pointed out, using different real number representations will result in different ground energies, depending on the capability of real number representations to represent real numbers. However, since the same linear equations are simply converted to QUBO Hamiltonians in different ways, these QUBO Hamiltonians solve the same original problem regardless of the real number representations. Therefore, there is no problem if different real number representations produce different QUBO Hamiltonians to examine the accuracy of the solution of the original linear equations. Rather, in our research, we mainly determined whether different real number representations may not be capable of taking full advantage of the inherent expressive power of real number representations owing to the differences in the ease of reaching the ground states. Our results, including those shown in Figs 2–7 in the revised manuscript, indicate that all the four real number representations can be best representations depending on the difficulty and size of the problem, regardless of the inherent expressive power of the real number representation.

We hope that the above has sufficiently answered your question.

[Comment 2-15]
Line 166; If I understand correctly, the selected matrix coefficients are positive random, real numbers (up to some precision). If that is correct, then I am very curious what the exact QUBO formulation is (e.g. item 10), because these generated QUBOs would, I think, be very complex in terms of range of coefficients and total number of variables. And, it seems like some matrix entries are also chosen which are negative - which makes me even more curious what the exact QUBO formulation is, since I am not sure that matrix factorization with negative coefficients can be formulated as easily as is shown in eq. 5.

[Response 2-15]

Thank you for pointing out your concern about the matrix generation for solving linear equations.

Please find that each element of matrix A generated by the process stated on line 166 in the original manuscript can be negative. For example, suppose

$$Q = \frac{1}{\sqrt{2}}\begin{pmatrix} 1 & -1 \\ 1 & 1 \end{pmatrix} \text{ and } \Lambda = \begin{pmatrix} 1 & 0 \\ 0 & -1 \end{pmatrix}$$

are generated as a result of QR decomposition of a random matrix and a random diagonal matrix, respectively. Then, we have

$$A = Q^T \Lambda Q = \begin{pmatrix} 0 & -2 \\ -2 & 0 \end{pmatrix},$$

which have negative elements.

We hope the above is a sufficient explanation of your question.

[Comment 2-16]
I suppose I would consider this a small comment, but it would be of interest to sample the problem QUBOs using classical heuristics as well, such as simulated annealing, steepest gradient descent, etc.

[Response 2-16]

Thank you for your constructive comments. As you pointed out, a similar situation occurs when using classical heuristics, and I believe that the results of Fixstars Amplify Annealing Engine, a GPU-based simulated annealing machine, are close to those of regular simulated annealing. On the other hand, the gradient method does not seem to be applicable to binary optimization problems. It is of course applicable to the least-squares form of the original linear equations, but that is not the purpose of this study, so I would like to omit it.

We hope the above has provided a sufficient explanation of your question.

# Grammar / Spelling / clarification Notes:

There are numerous grammatical errors throughout the manuscript that would need to be corrected before the paper is suitable for publication. Here are a few examples (this list is not comprehensive, and there are many more errors that need to be corrected):

[Comment 2-17]
Line 32. A potential fix would be to pluralize the phrase "quantum annealing machine"

[Response 2-17]

As you indicated, we have changed it to the plural form.

modified: on page 3, line 50.

From: quantum annealing machine

To: quantum annealing machines

[Comment 2-18]
Line 39. A potential fix would be “represented in their binary form”

[Response 2-18]

According to our research, leaving it as “represented in binary number” should be fine. Thank you for your suggestion.

[Comment 2-19]
Lines 56, 57, 58 do not make any sense - what does it mean to replace the objective function with the energy of the system? In quantum annealing specifically, the energy is the expectation value of the objective function, encoded using analog computation.

[Response 2-19]

Thank you for your comment. Certain physical phenomena have the property that an energy

function is defined for a state and the smallest energy state is realized with high probability. Therefore, by considering the energy function as an objective function, a solver that efficiently finds the minimum of the objective function can be used for physical calculations. An explanation of the above is given on lines 56–58. To further clarify the explanation above, we have revised the original text as follows.

modified: on page 3, line 55.

From: “If the objective function can be replaced with energy expressing the target phenomenon, quantum acceleration of finding of equilibrium state acquisition can be expected by a physics simulation [29,30,31,32]”

To: “Since some physical phenomena are simulated to find minimum energy states, quantum acceleration of physical simulations can be expected by giving the energy function as the objective function to the quantum solvers [34,35,36,37].”

[Comment 2-20]
Lines 60, 61, 62 state that: "The principle of operation is to find a solution to the variables by adjusting quantum fluctuations so that the objective function eventually becomes the global minimum". This is not accurate, and also does not make sense - what does it mean for the objective function to become the global minimum? The objective function just tells us what the cost value, or energy, or expectation value is of the objective function for a specific set of spin (or variable) states.

[Response 2-20]

We apologize for the misleading wording. As you pointed out, it is strange that the objective function becomes a global minimum. The correct expression here was to reach a state where the value of the objective function becomes a global minimum. We have corrected it as follows.

modified: on page 3, line 58.

From: “A quantum annealing machine specializes in combinatorial optimization problems. The principle of operation is to find a solution to the variables by adjusting quantum fluctuations so that the objective function eventually becomes the global minimum.”

To: “A quantum annealing machine specializes in combinatorial optimization problems. The principle of operation is to efficiently find the states in which the value of the objective function is a global minimum using quantum fluctuations.”

[Comment 2-21]
Line 66 states that "Many differential equations are derived from the energy functional based on the principle of least action" - what does differential equations implicately say about why quantum annealing makes sense as an analog computation for physical systems? This reasoning about why quantum annealing may be useful as an analog computation is not a correct summarization of how quantum annealing works.

[Response 2-21]

Quantum annealing machines can efficiently obtain states that minimize the value of the objective function. On the other hand, there are physical models that simulate by obtaining the state that minimizes the energy function. Here, if the energy function can be embedded as the objective function

of the quantum annealing machine, the property of the fast search of the quantum annealing machine may be used for fast physical simulations.

However, as you pointed out, this was not directly related to the principle of operation of quantum annealing machines. We would like to make the following minor corrections to avoid misunderstanding.

modified: on page 3, line 62.

From: “Physical simulations are one of the most promising practical applications of quantum annealing owing to their good compatibility with its operating principle. This is because many differential equations are derived from the energy functional based on the principle of least action, and the equilibrium state is calculated so that the energy of the system is minimized. Replacing the objective function with energy corresponds to solving a physical problem to find the equilibrium state of the system.”

To: “Physical simulations are one of the most promising practical applications because of their affinity with quantum annealing. This is because many differential equations are derived from the energy functional based on the principle of the least action, and equilibrium states are calculated so that the energy of the system is minimized. Regarding the energy of the system as an objective function, the physical problem of finding the equilibrium state of the system could be solved by a quantum annealing machine.”

[Comment 2-22]
Lines 62 and 63: "Since the Ising model is used as the basis, binary variables are used" - this is incorrect, an ising model corresponds to spin variable states not binary variables.

[Response 2-22]

As you pointed out, the Ising model is directly related to the spin state. However, it is true that spin states can easily be associated with binary variables; therefore, we have modified the text as follows:

modified: on page 3, line 60.

From: “Since the Ising model is used as the basis, binary variables are used.”

To: “Since the Ising models are used as the basis, they target objective functions with a combination of binary variables as the decision variables.”

[Comment 2-23]
Line 151: "the minimum Hamiltonian is explored using an Ising machine" I think the authors meant to say that the minimum variable assignment is computed using an Ising machine.

[Response 2-23]

There was an error as you indicated. We have made the following correction.

modified: on page 8, line 169.

From: “the minimum Hamiltonian is explored using an Ising machine.”

To: “the variable assignment that minimizes the value of the Hamiltonian is computed using Ising machines.”

Literature references:

[Comment 2-24]
There are several papers relevant to the topic of solving (or obtaining good solutions) types of linear algebra problems using quantum algorithms, including quantum annealing, that the authors do not cite or discuss.
1. https://arxiv.org/abs/2206.10576
2. https://arxiv.org/abs/1704.01605
3. https://arxiv.org/abs/2007.05565
4. https://arxiv.org/abs/2205.00645

Note that the generalization of matrix factorization is tensor factorization, the boolean version of which has also been investigated in the context of quantum annealing:
1. https://arxiv.org/abs/2107.13659
2. https://arxiv.org/abs/2103.07399
3. https://ieeexplore.ieee.org/document/9325388

The authors make some numerous observations about bit-flipping of some variable states on the problem Hamiltonians, including simultaneous bit flipping. This paper which investigates this topic in quantum annealing may be of interest: https://arxiv.org/abs/2210.16513

Lastly, it seems to me that this manuscript falls somewhat into the category of problem Hamiltonian encoding, and there are a couple of papers on ideas for other types of encodings:
1. https://arxiv.org/abs/1903.05068
2. https://ieeexplore.ieee.org/abstract/document/9951263/
3. https://arxiv.org/abs/2108.12004
4. https://arxiv.org/abs/2102.12224
6. PLOS authors have the option to publish the peer review history of their article ([a: https://journals.plos.org/plosone/s/editorial-and-peer-review-process#loc-peer-review-history]what does this mean?). If published, this will include your full peer review and any attached files.

[Response 2-24]

Thank you so much for taking the time to provide us with the important literatures that we had not fully grasped. We have carefully checked all the references you suggested and have properly cited them in the revised manuscript as follows.

(a) [https://arxiv.org/abs/2206.10576]: This paper estimates the required annealing time by simulating the time-varying process of the Hamiltonian in quantum annealing on a classical computer. Since this is closely related to Task 3, which is a newly added experiment in the revised manuscript, we have added the citation in the introduction of Task 3.

(b) [https://arxiv.org/abs/1704.01605, https://arxiv.org/abs/2007.05565, https://arxiv.org/abs/2107.13659, https://arxiv.org/abs/2103.07399,

https://ieeexplore.ieee.org/document/9325388]: These papers are studies of matrix decomposition with quantum annealing. Although our work is not directly related to matrix factorization, we have cited them in the introduction section because it is an important example of a calculation using quantum annealing.

(c) [https://arxiv.org/abs/2205.00645]: This paper is a study of the solving linear equations using gate-based quantum computers. Since the paper is related to solving linear equations, we have cited in the introduction section.

(d) [https://arxiv.org/abs/2210.16513]: This paper is a study showing that when using the reverse annealing method, starting with the initial state that has a small Hamming distance from the optimal state yields good results. Because of its relevance to our work in terms of the small number of bit flips, we have cited it in the introduction section discussing the relevance of bit flips.

(e) [https://arxiv.org/abs/1903.05068,

https://ieeexplore.ieee.org/abstract/document/9951263/,

https://arxiv.org/abs/2108.12004, https://arxiv.org/abs/2102.12224]: These papers are theoretical and experimental comparative studies on the representation of multivalued variables, such as domain-wall and one-hot representations. Since they are highly relevant to the representation of real-valued variables, which is the subject of our research, we have cited them and provided explanations in the introduction section.

With respect to the addition of the above references, the changes to the revised manuscript are as follows:

added: on page 19, line 423.

"Note that such analysis by simulating the annealing process has been done in previous studies, albeit in the context of reverse annealing [59]."

modified: on page 3, line 52.

From: "They are often applied to combinational optimization problems [21] involving the objective functions of the target such as community detection [22], traveling-salesman problem [23] and so on [24,25,26,27,28]."

To: "They are often applied to combinational optimization problems [21] involving the objective functions of the target such as community detection [22], the traveling-salesman problem [23], and so on [24,25,26,27,28,29,30,31,32,33]."

modified: on page 4, line 72.

From: "Some methods of solving a system of linear equations involving real numbers by quantum circuits have been studied [33,34,35]."

To: "Some methods of solving a system of linear equations involving real numbers using quantum circuits have been studied [38,39,40,41]."

added: on page 5, line 109.

"In addition, a previous study of the reverse annealing method [51] has shown that the number of bit flips between the initial state and the optimal state is correlated with the accuracy of the obtained solution, which is also the basis of this study to explore the real number representation."

added: on page 5, line 112.

"Several methods of representation for multivalued variables have been proposed in previous reports [52,53,54,55], showing report their advantages and disadvantages for these representations."

As a final note, we would like to express our appreciation for your careful recommendations and thorough review of our paper. If you have any additional concerns that we can address to improve our paper, please do not hesitate to let us know.
