# Supplementary material for: Novel real number representations in Ising machines and performance evaluation: Combinatorial random number sum and constant division
Source: PLoS One. 2024 Jun 13;19(6):e0304594. doi: 10.1371/journal.pone.0304594 (PMC11175401; doi:10.1371/journal.pone.0304594)
Supplement: Attachment — Submitted filename: RevLetter_plos2_fin_checked.docx [file pone.0304594.s002.docx]

Response to the Reviewers’ Comments

Ref. #.	PONE-D-23-25182R1

Title :	Novel real number representations in Ising machines and performance evaluation: Combinatorial random number sum and constant division

Author :	Katsuhiro ENDO, Yoshiki MATSUDA, Shu TANAKA and Mayu MURAMATSU

Responding to the review results, we have significantly revised the manuscript. We highly appreciate the reviewers’ valuable comments and suggestions for corrections, which make the quality of the paper improved.

Below, we provide our responses to the reviewers’ comments. Since one reviewer gave his/her own comments separately, we have prepared separate answers. For each comment, we first cited reviewers’ remarks for reference, and then described how we have responded to those. The responses were reflected in the revised manuscript, in which the corrected or newly added contexts were written in red.

We would be very grateful if our paper could be published in “PLOS ONE”

Best regards,

Katsuhiro ENDO, Yoshiki MATSUDA, Shu TANAKA and Mayu MURAMATSU

**[For Reviewer 2]**

[Comment 1]
\# Follow up from my previous comments

I believe that the authors have, with great detail, addressed the majority of the questions and comments -- and I think that the current manuscript is significantly clearer. The clarifications on the real number representations make much more sense now, and the descriptions of quantum annealing theory and implementation are much clearer.

- For the literature references I suggested, to be clear the reason I mentioned matrix factorization papers is because they are solving types of linear equations, granted typically the underlying decision variables are spins or boolean variables.

- I do not think the authors need to perform such simulations, but I mentioned steepest gradient descent as a possible additional classical heuristic - and it is true that gradient descent does not actually apply for these problems, but there does exist discrete steepest descent solvers: https://docs.ocean.dwavesys.com/projects/greedy/en/latest/

- Additionally, now that the authors have clarified that the GPU based simulator was in fact running simulated annealing, I am satisfied with this comparison.

[Response 1]

We thank the reviewer for taking the time to further review our manuscript and providing a critical assessment of our work. We also thank the reviewer for acknowledging most of our revisions to the previous peer review. Your comments, including those mentioned above, have deepened our peripheral knowledge of our research results.

Regarding the papers on matrix factorization that the reviewer mentioned in the previous comment, they certainly solve linear equations, although their decision variables are binary variables. In future research, we would like to investigate the effect of real number representations in such matrix decomposition methods when the variables are real numbers represented by binary variables. According to your comment, we modified the sentence “They are often applied to combinational optimization problems [21] involving the objective functions of the target such as community detection [22], the traveling-salesman problem [23], and so on [24,25,26,27,28,29,30,31,32,33].” to “They are often applied to combinational optimization problems [21] involving the objective functions of the target such as community detection [22], the traveling-salesman problem [23], matrix factorization [24,25,26,27,28] and so on [29,30,31,32,33].” on page 3, line 54.

Regarding the steepest descent solvers, as the reviewer stated, it is sure that a discrete analogue of the steepest descent algorithm such as dwave-greedy can be applied to our binary optimization problems. When the naive steepest descent solver is applied to the linear equation problems, the computational accuracy achieved by each real number representation will likely be similar to our results. That is, the CONSTANT representation will reach the best accuracy that the representation can reach, while the BINARY representation will get stuck in a local solution. This is because the steepest descent solver is a method that reaches a local optimal solution, and the binary optimization problems that require flipping multiple bits simultaneously to reduce the value of the Hamiltonian are likely to be very complex nonconvex functions. This explanation has been added shortly to the text as follows on page 5, line 95: “A similar property can be inferred for the greedy steepest descent algorithm [47], which is another classical method, because of its repeated local optimization.”

The distance here is the Euclidean distance between the real value which is expressed in the binary variables using the real number representation and the target real value.

We have responded to all comments we have received this time, and we hope that you will confirm the corrections.

[Comment 2]
\# Comments on the revised manuscript

1. An overall comment on the motivation and experimental description of the manuscript is that simulated annealing, and in general classical heuristics, are not constrained to perform only Hamming distance 1 (e.g. single bit-flip) local neighborhood searches. In particular, statements such as Line 351 where a single bit flip example is used, or Line 420: "bit-flip-based search, which mimics the process of simulated annealing" are not true that this type of search would mimic simulated annealing.

[Response 2]

Thank you for your comments regarding the simulated annealing method. As you pointed out, local neighborhood search with Hamming distance of 1 is not a strictly necessary constraint in simulated annealing. However, for example, in the case of the simulated annealing method that utilizes the Metropolis-Hastings algorithm (which is commonly used as an implementation of simulated annealing), if the state is changed drastically at a single step, the energy difference between the states before and after the change becomes too large, resulting in a very low probability of movement. For this reason, it is certain that local neighborhood search, in which only one part of the state is changed, is often employed for efficiency reasons.

In accordance with your comment, we have revised the manuscript. We added the following sentences on page 16, line 355:

In Tasks 1 and 2 we are considering the repeated process of moving to a such neighborhood. The local neighborhood search with Hamming distance of 1 is not a strictly necessary constraint in simulated annealing. However, for example, in the case of the simulated annealing method that utilizes the Metropolis-Hastings algorithm (which is commonly used as an implementation of simulated annealing), if the state is changed drastically at a single step, the energy difference between the states before and after the change becomes too large, resulting in a very low probability of movement. For this reason, it is certain that local neighborhood search, in which only one part of the state is changed, is often employed for efficiency reasons.

We also modified the sentences “In Task 1 and 2, we examined the efficiency of bit-flip-based search, which mimics the process of simulated annealing when using different real number representations.” to “In Tasks 1 and 2, we examined the efficiency of bit-flip-based search, which mimics the process of simulated annealing with local neighborhood searches when using different real number representations.” on page 20, line 434.

[Comment 3]

2. Line 84: "For example, consider the case where a single real number is optimized in a classical simulator": This example is unclear and should to be clarified -- what is being optimized? Is the real number the cost function?

[Response 3]

We apologize for the misleading wording which contained a grammatical error. The correct phrase is “Consider solving an optimization problem with only one real number as the decision variable”. Following your comment, we modified our manuscript. We modified the sentence “For example, consider the case where a single real number is optimized in a classical simulator of annealing machine.” to “For example, consider solving an optimization problem with only one real number as the decision variable in a classical simulator of annealing machine.” on page 4, line 84.

[Comment 4]

3. Line 637: "The error bars denote the standard error of the mean errors." I do not understand this measure -- do the authors intend to say that the errors bars denote the standard deviation of the distribution of the error measure for the repeated trials?

[Response 4]

Thank you for pointing out the part where the wording is not appropriate. This error bar shows the standard error of the estimated mean value of absolute error *E* (Eq. 11 in our manuscript). In this experiment, we have performed multiple trials with the same conditions for statistical analysis, and each trial returns a single error *E*. Based on the error *E* which varies stochastically in each trial, the mean value of error is estimated. The error bar shows the standard error, i.e., error of estimated value. The error bars in Fig. 2 are sufficiently small that the mean of the errors can be considered sufficiently reliable.

For better clarity, we have revised the text. We modified the sentence “The error bars denote the standard error of the mean errors.” to “The error bars denote the standard error of the estimated mean errors.” on page 31, line 653.

[Comment 5]

4. Line 357: "Then, we search for the state that is closest to the target value, limiting the path to only repeated transitions to states with decreasing distances that can be reached by the flipping of bits up to the": What is the distance being used here? Is it hamming distance?

[Response 5]

I apologize for not being clear about the distance in the part you mentioned. The distance here is the Euclidean distance between a real value, which is expressed in the binary variables using a real number representation, and a target value, which is also a real number. In other words, in Task 1 on line 357, one real value expressed in the binary variables using any of the four real number representations is allowed to move only in the direction closer to the target value. This explanation has been added to the text as follows on page 17, line 370:

The distance here is the Euclidean distance between the real value which is expressed in the binary variables using the real number representation and the target real value.

[Comment 6]

5. Line 395: "Fig 5 shows the results of the task, where each subplot in the figure corresponds to a different condition"; is this referring to each subplot showing results for a different condition number? Or, different parameter combinations? From looking at Figure 5, it does not appear that the condition number is mentioned in the plots.

[Response 6]

Thank you for pointing out the unclear statement in the relevant section. Fig. 5 shows the results of the 10000 trials each with different values of *K* (the number of steps) and *R* (the number of bits). Each subplot in Fig. 5 (except for the statistical summary on the left) corresponds to the results of specific combinations of *K* and *R*. In other words, the "different condition" in L. 395 refers to a variation in both *K* and *R*. The values of *K* and *R* for each subplot are listed in the subtitle at the top of each subplot.

Note that in Task 2, which includes L. 395, there is no condition number because we are considering a simplified problem with only one real number as the decision variable. In response to your comment, we have revised the text as follows to clarify exactly what a "different condition" is. We added the following sentence on page 17, line 376: The condition of each subplot (the values of *K* and *R*) is listed in the subtitle at the top of each subplot.

[Comment 7]

6. There are still a few grammatical errors throughout the text that should be fixed.

[Response 7]

We apologize for the remaining grammatical errors. We have sent the manuscripts to English proofreading services, and the revised manuscript reflects the corrections.

As a final note, we would like to express our appreciation for your careful recommendations and thorough review of our paper. If you have any additional concerns that we can address to improve our paper, please do not hesitate to let us know
